# Silicon as Versatile Player in Plant and Human Biology: Overlooked and Poorly Understood

**DOI:** 10.3389/fpls.2015.00994

**Published:** 2015-11-12

**Authors:** Muhammad Ansar Farooq, Karl-Josef Dietz

**Affiliations:** Department of Biochemistry and Physiology of Plants, Faculty of Biology, University of BielefeldBielefeld, Germany

**Keywords:** silicon, dietary sources, human exposure, health benefits, plant nutrition, stress tolerance

## Abstract

Silicon (Si) serves as bioactive beneficial element. Si is highly abundant in soil, and occurs ubiquitously in all organisms including plants and humans. During the last three decades, nutritional significance of Si for plant and human health has received increasing attention. Plant Si plays a pivotal role in growth and development, and this beneficial effect depends usually on accumulation in plant tissues, which are then protected from various forms of biotic and abiotic stresses. Likewise, human exposure to Si imparts health benefits and essentially occurs through plant-derived food products. Si bioavailability in human diet, e.g., strengthens bones and improves immune response, as well as neuronal and connective tissue health. Despite this empiric knowledge, the essentiality of Si still remains enigmatic. Thus the link between Si availability for plant development and its profound implication for human welfare should receive attention. This review aims to provide a broad perspective on Si as important element for plant and human nutrition and to define research fields for interdisciplinary research.

## Introduction

Silicon (Si) is omnipresent and takes part in global biogeochemical Si cycles, both in oceans and on terrestrial areas (Basile-Doelsch et al., 2005). Until 1960s, the essentiality of Si was best known for lower forms of life, in particular diatoms, sponges and corals. Si is required for normal cell growth and imparts structural benefits to diatoms, radiolarians, and some sponges (Carlisle, 1997). However, Si also cycles between plants and the environment thereby realizing multiple functional benefits. Ferns and many monocots accumulate Si to high amounts (Hodson et al., 2005). Thus functional significance of Si in modulating growth performance and ameliorating stress in higher plants is widely accepted. This view is based upon a range of field and laboratory experiments indicating that Si serves manifold roles in plants (Epstein, 1999, 2009; Rafi and Epstein, 1999; Rains et al., 2006; Ma et al., 2011).

The ample Si supply from soil to plants exceeds uptake of essential nutrients in several species including cereals (Epstein, 1994). Plants grown under natural conditions are exposed to diverse biotic (diseases caused by viral and bacterial pathogens or fungi and herbivores) (Miyake and Takahashi, 1983; Cherif et al., 1994; Savant et al., 1997) and abiotic (salinity, heat, cold, wind, water, and mineral deficiency or excess) stresses, often in combinations (Ma et al., 2001; Ma, 2004; Farooq et al., 2015). Thus plants face an enormous combinatorial complexity with basically infinite environmental conditions. Si enhances physical and chemical defense power of plants (Epstein, 1999). However, beneficial effects of Si are most obvious in high Si-accumulating plant species (Ma et al., 2011). Investigation of crop species of the Si-accumulating type including cereals revealed an active mode of Si uptake and transport system, which enable them to realize the high Si requirements of their plant body (Ma et al., 2006, 2011). On the other hand, Si deficiency does not interrupt the life cycle in plants, therefore its absolute requirement and essentiality continues to be debated (Marschner, 1995). During recent years along with the growing interest of plant biologists to understand Si dependencies and anomalies in plants, the nutritional function of Si has also received attention in human biology, where equally important features have been established by now. Silica is prevalent in the typical human diet with concentrations tending to be much higher in plant based foods and has a multitude of uses, e.g., strengthen bones and connective tissues, reduces risks of alopecia, Alzheimer’s and cardiovascular diseases (Jugdaohsingh et al., 2000; Jugdaohsingh, 2007; Nielsen, 2014). Thus Si plays a significant role in modulating physiological and metabolic responses both in plant and human biology. Most of the previously published reviews focused on either aspect of nutritional significance of Si (Guntzer et al., 2012; Wu et al., 2013; Nielsen, 2014; Zhu and Gong, 2014; Pontigo et al., 2015). There is a need for comprehensive information covering the breadth and versatility of silica. Therefore, the intent of this review is to critically evaluate both types of evidences available to support the nutritional significance of silica for plant stress tolerance and health benefits of dietary silica primarily derived from plant-based foods. The article compiles the present state of knowledge on the availability, uptake, distribution and positive potential of Si, but also shows that the molecular understanding of involved mechanisms of beneficial action still awaits clarification which is critical to fostering the much needed research at interdisciplinary level. Further, the information from this review shall be used to develop strategies to manipulate plant silica contents for enhanced plant tolerance against various kinds of environmental stresses and improved nutritional quality for human health.

## Good Nutritional Diet Begins with the Soil

**FIGURE 1:**
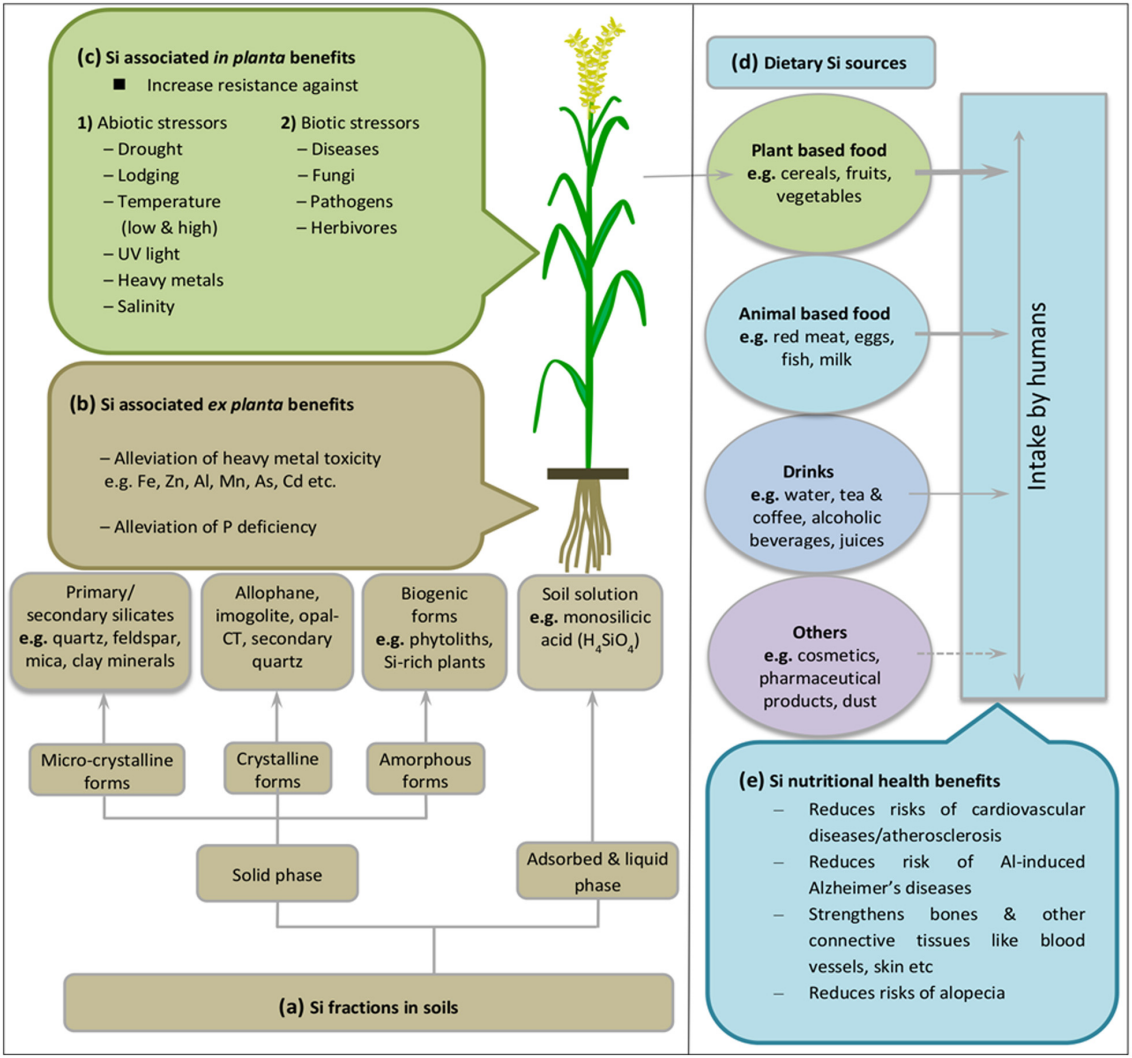
**Overview of the Si cycle between soil, plants, and its subsequent intake by humans. (a)** Classes of Si compounds in soil, modified with permission from Sauer et al. (2006). Weathering of silicate-containing minerals releases Si as silicic acid into soil solution, which is passively or actively taken up by plant roots (cf. **Figure 2**, active uptake and transport mechanisms). Presence of Si both in soil **(b)** and plants **(c)** protects plants against biotic and abiotic stresses. **(d)** Dietary Si sources for human intake, with maximum contribution from plant-based food products as represented by arrow thickness (see also **Table 1**). Broken line indicates very low level of contribution. In **(e)** nutritional benefits of Si for human health are listed. See main text for more details.

Silicon is the second most abundant element in the earth crust with a mean share of 28.8% (dry weight basis) (Epstein, 1999) mostly ranging between 50 and 400 g Si kg^-1^ of soil (Kovda, 1973). Generally, Si compounds exist in different soil fractions such as solid, adsorbed or liquid phases (Sauer et al., 2006) (**Figure 1a**). The pre-dominant Si forms in mineral soils include silica (SiO_2_), and primary (e.g., quartz, feldspar, mica) or secondary (e.g., clay minerals) silicate minerals which contain Si, oxygen, and metals like Al (aluminosilicates) and Mg (talc) (Farmer et al., 2005; Rezanka and Sigler, 2008). SiO_2_ comprises up to 45% of soil mass and represents >95% of the secondary Si-enriched horizons (Summerfield, 1983). Additionally, Si compounds exist in various amorphous forms of biogenic origin such as phytoliths and silica rich plants (Cornelis et al., 2011). The biogenic silica contributes 1–3% of total Si pool in soil (Desplanques et al., 2006).

Weathering of silicate-containing minerals is the major source of chemical elements for terrestrial plants, and, thus for the whole nutritional chain. The mineral breakdown releases soluble silica mainly as silicic acid (H_4_SiO_4_) into the soil solution (liquid phase), surface water and other natural water bodies with variable contents of 0.1–0.6 mM. This is about twofold the average soil phosphorous contents and similar to macro-nutrients such as calcium, potassium, and sulfur (Epstein, 1994). However, the dissolved Si concentration varies considerably depending on the type of minerals and the biotic and abiotic environment (Datnoff et al., 2001; Guntzer et al., 2012). Silicic acid is weakly acidic (*pKa*_1_ = 9.70 and *pKa*_2_ = 12) and below pH 9 it commonly occurs as uncharged monomeric form [(H_4_SiO_4_)^o^] which is the most readily absorbable form of Si in humans and plants (Weast and Astle, 1983; Knight and Kinrade, 2001; Jugdaohsingh et al., 2002; Ma et al., 2008).

Some of the released silica is adsorbed to soil minerals such as Fe and Al oxides/hydroxides (Dietzel, 2002) and competes with other anions for sorption sites (**Figure 1a**). Despite the fact that most soil reservoirs are rich in Si, plant-available Si may be limited depending on soil type and seasonal changes. Young mineral soils which are less weathered usually supply more Si than completely weathered acidic soils to the biosphere (Skjemstad et al., 1992).

## Ex Planta Benefits of Silicon Relate to Abundance in Soil

Silicon release from weathering of silicate-containing minerals activates plant acclimation responses against multiple abiotic stresses. Improved stress tolerance has partly been related to silica presence in the soil inducing *ex planta* benefits in the rhizosphere and to the silica pool that has entered the plant body causing positive *in planta* effects (**Figures 1b,c**). Indigenous Si pools or artificial amendment of soils with silicate-containing fertilizers affect soil properties and improve availability of essential elements such as phosphorous (Fischer, 1929; Brenchley et al., 2008). In addition, Cheong and Chan (1973) reported that the beneficial effect of Si under phosphorous deficiency is attributed to increased levels of organic phosphoesters, thereby improved utilization of phosphorous inside plant body. Later on Eneji et al. (2008) confirmed the correlation between phosphorous availability and Si presence outside the plant tissue and concluded that Si fertilization improves phosphate availability to plants in low phosphorous soils. Interestingly the opposite response was seen under conditions where excess phosphorous was applied. Then Si application reduced toxic effects of phosphorous by limiting its availability, and ultimately reduced chlorosis (Ma et al., 2001). Thus Si establishes a phosphorous buffer system.

Similarly, soil Si immobilizes toxic metal ions such as alumi num (Al), arsenic (As), cadmium (Cd), iron (Fe), manganese (Mn), and zinc (Zn) via complexation, ultimately removing them from the rhizosphere as insoluble precipitates (Liang et al., 2005; da Cunha et al., 2008; Naeem et al., 2014). For instance, Si forms complexes with Al creating inert hydroxyl-aluminosilicates (HAS) in soil solution and reduces bioavailability of toxic Al ions (Hodson and Evans, 1995; Li et al., 1996; Liang et al., 2007). In maize, Si stimulates root exudation of phenolic compounds which form complexes with Al ions and reduces their uptake by plant roots (Kidd et al., 2001). Additionally, Si in the growth media ameliorates As toxicity in rice. Both Si and As share common root uptake and transport pathway. Therefore elevated Si abundance in the soil solution reduces As uptake and subsequent accumulation in rice shoots (Ma et al., 2008). Further, exogenous application of Si increases soil pH and decreases solubility and thus availability of toxic metals. For example, addition of furnace slag as Si source in paddy field reduced Cd uptake possibly by increasing soil pH and subsequently also root to shoot translocation (Shi et al., 2005; Liang et al., 2007; Lu et al., 2014). However, an alternative explanation was proposed by da Cunha et al. (2008) who found that applied calcium silicate reduced Cd and Zn concentrations in maize shoots by changing metal ion speciation in the soil solution without affecting soil pH. Similarly, the beneficial effect of Si application to reduce Mn toxicity is attributed to enhanced Mn deposition in the cell wall and hence reduced uptake in the cytoplasm (Rogalla and Romheld, 2002; Wiese et al., 2007). Likewise, Ma and Takahashi (2002) found that Si application reduced Fe toxicity in rice. Under such conditions, oxidative activity of rice roots increased by Si fertilization, thereby stimulating conversion of Fe^2+^ (ferrous; soluble form) to Fe^3+^ (ferric; insoluble form). This process resulted in the precipitation of Fe in the growth media or at the root surface (iron plaque; Fu et al., 2012) and ultimately reduced Fe uptake and toxicity in plants. All these reports highlight the importance of bioactive silica in soil reservoirs which interact with toxic metals and reduce their availability by increasing soil pH, metal immobilization in the growth media and also by changing metal distribution inside the plant.

Contrarily, the beneficial role of Si under metal deficiency conditions has also been recently assessed in several plant species (Gonzalo et al., 2013; Pavlovic et al., 2013; Bityutskii et al., 2014). Generally, immobilized metal pools formed under both metal toxic and non-toxic conditions are known to serve as source for plant nutrition through remobilization during micronutrient deficiency periods (Bienfait et al., 1985; Briat et al., 1995; Waters et al., 2009). As discussed above, the formation of Fe, Mn, and Zn deposits in the cell wall of roots by the application of Si under metal toxic conditions provide general evidence in this regard (Rogalla and Romheld, 2002; Wiese et al., 2007; da Cunha et al., 2008; Fu et al., 2012). However, the effect of Si addition was further investigated under Fe deficiency in cucumber and soybean (Gonzalo et al., 2013; Pavlovic et al., 2013; Bityutskii et al., 2014). The results demonstrated that total plant Fe contents did not improve significantly by pre-application of Si during sufficiency period, while more Fe accumulated in roots of Si-treated cucumber and soybean plants, owing to its precipitation at the root surface and high Fe accumulation in the root apoplast (Pavlovic et al., 2013). Subsequently, root Fe apoplastic pool decreased dramatically after the first day of Fe deficiency until complete depletion of Fe in the root apoplastic pool within next 5 days. In contrast, Fe concentration in the xylem sap and its subsequent distribution in plant body improved significantly by the supplementation of Si to Fe-deficient plants. Under such conditions, citrate production in xylem sap, root, and leaf tissues of Si-treated cucumber plants increased significantly, thereby facilitating long distance transport of Fe through the xylem and improving utilization in leaves (Rellán-Alvarez et al., 2010; Pavlovic et al., 2013; Bityutskii et al., 2014). Apparently Si application promotes Fe storage in the root apoplast during sufficiency/excess toxicity periods. Fe remobilization during deficiency periods seems to be the main factor contributing to the beneficial effect of Si under Fe deficiency conditions. Similarly under Mn and Zn toxicity, enhanced metal immobilization in root cell wall due to presence of Si are reported in several studies (Currie and Perry, 2007; Gu et al., 2012), while little is known about their occurrence under normal growth conditions and the beneficial role of Si in their remobilization when required (Bityutskii et al., 2014). Nevertheless under Zn deficiency, production of citrate increased in roots of Si-treated cucumber plants which could improve Zn distribution as described before for Fe-deficient plants (Bityutskii et al., 2014). However, further research efforts coupled with advanced methodological approaches are required to evaluate the potential of Si in alleviation of micronutrient deficiency in crop plants.

## From Soil to Plant: Silicon uptake and Transport Mechanisms

Plants accumulate silica to 0.1–15% of their dry weight. The degree of accumulation depends on uptake and transport mechanisms which differ significantly between species (Takahashi et al., 1990; Ma et al., 2001). Strong genotypic differences are reported even within species. Following the discovery of Si transporters in rice, over 500 plant species have been studied for their Si uptake and transport mechanisms and were placed into different categories depending on their silica contents (Ma and Takahashi, 2002). Among them, monocots such as rice, wheat, maize and barley are categorized as Si accumulators due their very high silica contents (10–15%) (Hodson et al., 2005; Ma and Yamaji, 2015). Interestingly other monocots including also distinct members of the orders of Poales and Aricales and most dicots accumulate less than 0.5% Si in dry mass (Neumann, 2003; Ma et al., 2008). Thus at least for Si-accumulators the Si uptake exceeds that of macronutrients essential for plant growth and development.

**FIGURE 2:**
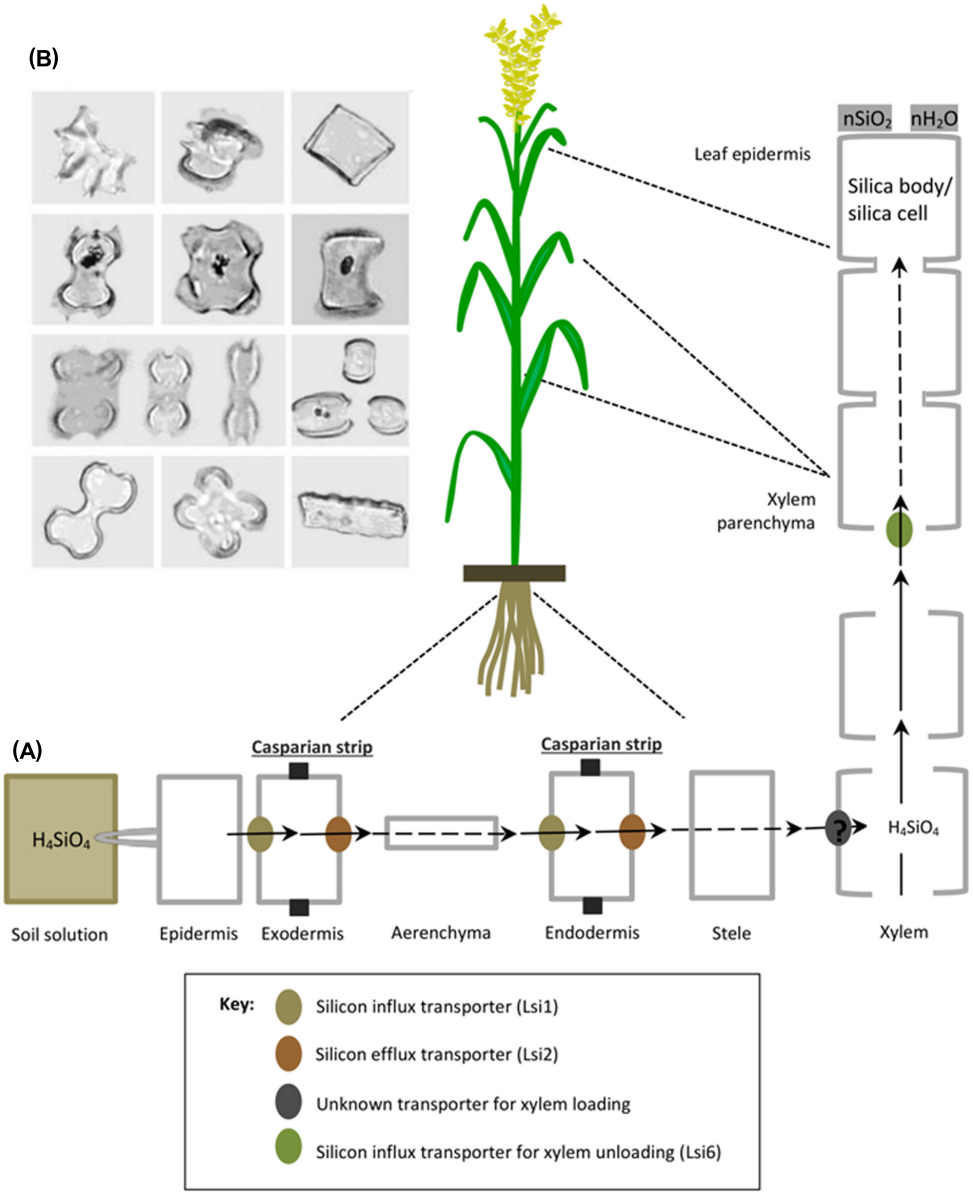
**A schematic representation of silicon uptake, transport and accumulation in rice. (A)** Rice roots absorb silicon (as silicic acid) from soil solution, which is transported to the root exodermis by influx transporter (Lsi1) and subsequently released to the apoplast of the aerenchyma by an eﬄux transporter (Lsi2). Successively, it is transported into root endodermis by Lsi1 and released to the stele by Lsi2. Then silicic acid is loaded into xylem by an unknown transporter and translocated to the shoots via the transpiration stream. In leaves, silicic is unloaded by another influx transporter (Lsi6), localized in the xylem parenchyma cells of leaf sheaths and leaf blades. In shoots and leaves, silicon is transformed from aqueous form (silicic acid) to solid amorphous silica (SiO_2_–nH_2_O) called silica bodies/silica cells and deposited mainly in the cell walls of different tissues such as leaf epidermal cells. Reproduced with permission from Ma et al. (2011), copyright Proceedings of the Japan Academy, Series B. **(B)** Various morphological shapes of silica bodies detected in leaves of different grass families, reproduced with permission from Piperno and Sues (2005).

Silicic acid (H_4_SiO_4_) dissolved in the soil solution is taken up by plant roots as an uncharged monomeric molecule below pH 9 and subsequently transported through the roots either actively in an energy-dependent process or passively (energy-independent process) which occur against and down an (electro-)chemical potential gradient, respectively. The active mode of uptake is mostly dominant in certain monocots such as rice, wheat, maize and barley (Takahashi et al., 1990; Ma et al., 2001, 2006, 2007a, 2011; Mitani et al., 2009) and is characterized by the presence of influx and eﬄux transporters regulating optimum supply of Si to different plant tissues. For example in rice, a channel type influx transporter named Lsi1 (Low Si 1) mediates passive transport of Si across the plasma membrane between the external solution and the plant cells (**Figure 2A**). Lsi1 displays high sequence similarity with nodulin-26 like intrinsic proteins (NIP), a subfamily of plant aquaporins and is mainly localized in mature plant roots at the distal side of both exodermis and endodermis, where apoplastic barriers decrease free diffusion or mass flow (Ma et al., 2006) (**Figure 2A**). Although Lsi1 is a bidirectional passive channel, it functions as an influx transporter of Si in plant, because it cooperates with the eﬄux Si transporter named Lsi2 which is driven by the proton gradient across the plasma membrane (Ma and Yamaji, 2015). In contrast to Lsi1, Lsi2 facilitates active transport of Si out of the plant cells and is localized to the proximal sides of exodermis and endodermis and belongs to a family of putative anion transporters (Ma et al., 2007b). Therefore once absorbed by plant roots, subsequent silica transport from root cortex to stele is carried out by high affinity eﬄux transporter called Lsi2 (Low Si 2). Subsequently, Si as silicic acid is transported to the shoots via the transpiration stream in xylem. A transporter responsible for xylem loading of Si has not been identified yet. However, a transporter named Lsi6 (Low Si 6) was localized at the adaxial side of xylem parenchyma cells of leaf blades and leaf sheaths and found responsible for xylem un-loading (Yamaji et al., 2008). It also mediates preferential distribution of silicic acid to panicles by controlling inter-vascular transport in nodes (Yamaji and Ma, 2009). Until recently, many plant species such as rice, maize, barley, and wheat, and also recently some dicots like soybean and pumpkin have been studied and identified with efficient Si uptake and transport system (Mitani et al., 2009, 2011a,b; Chiba et al., 2009; Montpetit et al., 2012; Deshmukh et al., 2013). Plants with active uptake mechanism significantly decrease the free silica concentration of the soil solution. And in most of them, a major proportion of absorbed silica is translocated to above ground plant tissues where inorganic amorphous oxides of silica cristallize upon loss of water and accumulate extracellularly or intracellularly in plant body as solid silica bodies, silica cells or phytoliths [phyto means plant and Greek word lithos means rock] (Ma and Yamaji, 2006) (**Figure 2B**). According to an estimate, as much as 90% of total Si uptake are deposited in the cell wall of hulls and leaf epidermal cells and constitute up to 10% of dry weight in grass shoots (Yoshida, 1965; Ma and Takahashi, 2002; Raven, 2003). Other cellular compartments such as short and long cells of leaf epidermis, bulliform cells and dumbbell-shaped cells also contain silica. Intracellular accumulation of silica in cell cytoplasm and vacuole is generally stable even after plant decomposition and are found abundantly in soils with variable but distinct shapes like boats, bowls, dumbells, saddels etc. (Piperno, 1988; Lins et al., 2002) (**Figure 2B**).

## Silicon in Plants: the Stress Reliever

Until the advent of accumulating dioxygen about two billion years ago, the evidence of life involvement in the processing of silicic acid to biogenic forms such as phytoliths is totally lacking. However, today, plants and other biological organisms are known to incorporate silica in amounts of gigatons per year (Piperno, 1988). Biological membranes display a basal permeability to silicic acid (permeability coefficient ∼10^-10^ m s^-1^; Raven, 2001). Silicic acid moves across the membranes by dissolving in the lipid phase of the membrane in a process called ‘lipid solution’ transport (Raven, 2001). As a small, uncharged, monomeric molecule, silicic acid closely mimics water in its movement into and out of biota (Exley, 2009). However, the uptake and distribution of silicic acid by plants is increased many folds by the presence of different type of influx and eﬄux transporters (Ma and Yamaji, 2015). As pointed out above, absolute requirement and essentiality of Si for plants is still debated despite its extraordinary abundance in the earth and high biological availability. Most current knowledge about the utility of Si comes from biosilicifier species, in which silicic acid is deposited as amorphous silica (Perry, 2009). In general, biochemical functions of Si for plants can be attributed to aqueous silicic acid and physical functions to silica accumulated in solid form such as phytoliths.

Insight into Si functions in plant biology emerges from agricultural experiments conducted on higher plants, where Si supplementation significantly ameliorated a range of biotic and abiotic stress symptoms (**Figure 1c**). Therefore, use of silicate-containing fertilizers in agricultural crop production system is increasingly practiced to improve plant performance by alleviating stresses (Datnoff et al., 2001). It should be noted that Si-free growth of plants is difficult to realize due to Si contaminations in glassware, water and chemicals. Thus most studies compare highly Si-depleted growth conditions with Si-enriched conditions.

## Biotic Stresses

Silicon alleviates detrimental effects of biotic stresses through a range of mechanisms, including the production of anti-bacterial and antifungal compounds as a broad response against pathogen attack (Cherif et al., 1994). For instance, Si-induced resistance to powdery mildew in wheat and to blast in rice is attributed to enhanced production of antifungal compounds called phytoalexins (Rodrigues et al., 2003; Remus-Borel et al., 2005). Additionally, deposition of silica at the site of attack also reduces fungal and pathogen damage, owing to surface hardness which deters pathogen penetration (Piperno, 1988). Heine et al. (2007) reported that application of Si in bitter gourd, a moderate Si-accumulator, reduced the spread of root rot pathogen, but not in tomato, a low Si-accumulator. This indicates that fitness benefits involving Si vary between plant groups depending on Si-uptake and deposition mechanisms, potentially giving some plants an adaptive advantage in a diverse system.

Deposition and concentration of silica bodies in plant tissues such as wood, leaves and seed coats increase strength and rigidity of cell walls and provide resistance against herbivory attack, owing to their ability to wear down tooth enamel and ultimately compromised growth and reproduction of herbivore due to reduced nutrient and carbohydrate acquisition. Therefore silicate fertilization correlates with efficient defense mechanisms against feeding guilds by reducing palatability and digestibility of plants tissues (Skinner and Jahren, 2004; MacFadden, 2005). Cid et al. (1989) reported that leaf silicification is induced upon herbivory attack, with response patterns that were specific to herbivore type and the amount of damage sustained. For example, in areas under intensive grazing, silicification of grasses was reported to be greater (McNaughton et al., 1985).

## Abiotic Stresses

Silicon application also counteracts various kind of abiotic stresses including physical stress (drought, lodging, low and high temperature, UV light) and chemical stress (heavy metal and salinity) (Ma et al., 2006; Liang et al., 2007; Li et al., 2008) (**Figure 1c**). As described above, strengthened physical defense is attributed to presence of silicified structures called phytoliths or silica bodies. Increased thickness of leaf surfaces due to silica deposition underneath the cuticle reduces water loss by transpiration under drought stress (Hodson et al., 2005). Similarly, silica bodies accumulating in cell vacuoles reduce crop damage due to lodging, or improve light capture characteristics by keeping leaf blades erect, thus aiding photosynthetic process (Ma et al., 2011). In addition, silica bodies reduce leaf heat-load through efficient far-infrared thermal emission of silica providing a passive cooling mechanism under conditions of high solar irradiation (Wang et al., 2005).

The presence of silicic acid in dissolved form increases plant tolerance against salinity and metal ions such as Zn, Al, Mn, and Cd and is accompanied by increased activities of enzymatic antioxidants such as superoxide dismutase (SOD) and catalase, and non-enzymatic antioxidant (ascorbate) and reduced rate of lipid peroxidation (Neumann and zur Nieden, 2001; Zhu et al., 2004; Shi et al., 2005; Moussa, 2006). It should be noted that these observations describe secondary effects of the beneficial action of Si but unlikely address the primary mechanisms. Likewise, increased deposition of Si in plant roots reduces apoplastic flow and uptake of toxic metals (Ma et al., 2011).

The results summarized so far indicate that optimizing silica content in plants might be a promising strategy to increase general tolerance against multiple biotic and abiotic stresses. Frequently, the studies provide convincing but more descriptive evidence for the beneficial effect of Si and assess accompanying phenomena such as strengthened antioxidant defense and lowered oxidative damage. However, the molecular mechanisms of Si-dependent fortification against the stresses await elucidation, e.g., Si-dependent regulation of intracellular ion homeostasis or elevated chaperone levels. Future studies should take advantage of available genome-wide methods such as transcriptomics and proteomics to address changes in an untargeted manner and kinetically dissect first transcriptional responses and subsequently identify involved primary signaling and important metabolic pathways in combinatory stress experiments.

## Potential of Phytoliths for Carbon Sequestration

The industrial and technical revolution seriously threatens stability of the global climate due to increasing load of the atmosphere with CO_2_ and global warming. Plants play a fundamental role in the regulation of atmospheric CO_2_, and studies have established a significant relationship between silicate weathering and CO_2_ consumption (Li et al., 2011; Song et al., 2011). The high nutrient requirement of vascular plants is covered by active mobilization of nutrient reserves. This is achieved by root-induced acidification, activation of chelation mechanisms and shifts in ion exchange equilibria. All these processes alter the soil physical properties and cause a continued soil development which accelerates the process of silicate weathering. As a result, CO_2_ is consumed in a reaction where silicic acid is released during the breakdown of calcium and magnesium containing silicate minerals (Struyf et al., 2009). Gaillardet et al. (1999) reported that annually 0.104 Gt carbon is consumed during the process of silicate weathering which indicates a crucial role of plants in the terrestrial biogeochemical cycles of Si with significant implications on the global carbon cycle (Serna and Fenoll, 2003). Inside the plant body, silicic acid is polymerized to form silica bodies/phytoliths which encapsulate carbon macromolecules and cellular organelles such as plastids and mitochondria. These phytoliths are entrapped within cell vacuole (Piperno, 1988; Raven et al., 1999; Ma, 2003; Neumann, 2003; Carter, 2009). Phytolith morphologies can be diagnostic of plant species (Piperno, 1988) and usually consist of 66–91% silica, but also 1–6% occluded carbon, iron and aluminum (Wilding, 1967; Parr et al., 2010). After decay of the organic plant matter, phytoliths are released into the soil and may remain stable for many years, thereby increasing the chemical and physical protection of organic carbon. However, these properties greatly vary with phytolith composition, soil chemistry, and climatic factors (Alexandre et al., 1997; Blecker et al., 2006). According to an estimate, phytoliths mediate carbon sequestration of Si-accumulating crop species and bamboo in the range of approximately 1.5 billion ton-equivalents-CO_2_ per year, which equates to sequestering 11% of the current increase in atmospheric CO_2_ (Parr et al., 2010).

## Silicon in Humans

In recent years, research focuses on the effects of silica on human health, in contrast to prior research which focused solely on nutritional significance of Si for plant growth and development. Plant-based foods, however, are the major contributor to dietary silica, or Si. This includes cereal grains and grain products, vegetables and beverages (**Table 1**; detail discussed in the next section) (Pennington, 1991; Powell et al., 2005). Circumstantial evidence for the essentiality of Si in animals, the presence of silica in most cells and in primitive organisms such as bacteria, viruses, and fungi suggest that silica may have a desirable or even an essential role in all organisms (Schwarz and Milne, 1972; Iler, 1979). Si is actively up-taken and transported by diatoms, algae, and sponges and is essential for their survival and replication (Werner, 1977; Iler, 1979). In 1972, Si deprivation experiments by Carlisle and in parallel Schwarz and Milne on growing chicks and rats suggested that dietary Si is essential for the normal growth of chicks and rats. Si deficiency in their diet caused abnormal tissue growth, particularly of collagenous tissues such as skull and peripheral bones, joints, hair, and skin. Both studies suggest that silica may also be essential for higher animals, including humans. They mark the beginning of bio-Si research and its significance for human health, especially in orthopedics (Hench and Wilson, 1986; Kokubo et al., 2004; Jugdaohsingh, 2007). Globally, osteoporosis is a growing health problem which is caused by low mass and increased fragility of bones, and leads to severe disabilities and elevates mortality rates. Annual costs of treating osteoporosis exceeds one billion pounds in countries like UK and requires cogent, long-term preventive strategy (Christodoulou and Cooper, 2003; McClung, 2003). Subsequently, many human nutritionists focused on the understanding of dietary importance of Si on bone health. Relevant data on Si sources for human intake and associated health-associated benefits are compiled in the next sections.

**Table 1:** Dietary Si sources and their contribution for human intake.

	Plants	Human intake		
Sources	Shoot Si conc.	Food product	Food Si content	Reference
	(% DW)	(ingested)	(mg/100 g)	
**Plant based**				
Rice	8.0	Chocolate covered cereal	5.02	Hodson et al., 2005; Powell et al., 2005
		Boiled (basmati/brown/white)	0.98–3.76	Hodson et al., 2005; Powell et al., 2005
		Toasted and crisped cereal	3.13	Hodson et al., 2005; Powell et al., 2005
Wheat	2.46	Cereal biscuits	5.25	Hodson et al., 2005; Powell et al., 2005
		Flakes	4.60	Hodson et al., 2005; Powell et al., 2005
		Flour (whole meal/white)	3.04–4.29	Hodson et al., 2005; Powell et al., 2005
		Bread	3.5	Varo et al., 1980a; Hodson et al., 2005
Sugar beet	2.34	Fresh	21	Kaufmann, 1993; Draycott, 2006
Oats	2.04	Roasted meal	260	Varo et al., 1980a; Kaufmann, 1993
		Oatcakes	18.26	Kaufmann, 1993; Powell et al., 2005
		Bread	7	Varo et al., 1980a; Kaufmann, 1993
		Rolled, cooked	1	Varo et al., 1980a; Kaufmann, 1993
Barley	1.82	Roasted meal	240	Varo et al., 1980a; Hodson et al., 2005
		Malt	210	Varo et al., 1980a; Hodson et al., 2005
		Flakes	9	Varo et al., 1980a; Hodson et al., 2005
		Boiled	1.84	Hodson et al., 2005; Powell et al., 2005
Rye	1.58	Malt	11	Jones and Handreck, 1967; Varo et al., 1980a; Kaufmann, 1993
		Rye meal	8	Jones and Handreck, 1967; Varo et al., 1980a; Kaufmann, 1993
		flour	7	Jones and Handreck, 1967; Varo et al., 1980a; Kaufmann, 1993
		Flakes	4	Jones and Handreck, 1967; Varo et al., 1980a; Kaufmann, 1993
Soybean	1.40	Dried and boiled	1.19	Hodson et al., 2005; Powell et al., 2005
Maize (Corn)	0.83	Flakes	4	Varo et al., 1980a; Kaufmann, 1993; Hodson et al., 2005
Beans (green)	–	Fresh, boiled	5–8	Powell et al., 2005
Red beet	–	Fresh, raw	25.4	Haghiri, 1964
Carrot	–	Fresh, raw	17	Whitton and Wells, 1978
Potatoes	0.40	Mashed, powder	2	Varo et al., 1980b; Kaufmann, 1993; Hodson et al., 2005
Asparagus	0.27	Fresh, canned	3	Varo et al., 1980b; Kaufmann, 1993
Lettuce	–	fresh, raw	2	Varo et al., 1980b; Pennington, 1991
Banana	–	Fresh, raw	8	Varo et al., 1980b; Pennington, 1991
Apple	–	Fresh, raw	0.2–0.5	Varo et al., 1980b; Pennington, 1991
Nuts	–	Roasted	0.2–0.6	Powell et al., 2005
**Animal based**				
Eggs	–	Boiled, cooked	2–4	Nielsen, 1974; Varo et al., 1980c
Red meat	–	Cooked	0.5–2	Nielsen, 1974; Nuurtamo et al., 1980
Fish	–	Cooked	0.5–1	Nielsen, 1974; Nuurtamo et al., 1980
Milk	–	Fresh, buttermilk, Yogurt	0.14–0.48	Grebennikov et al., 1964; Nielsen, 1974; Varo et al., 1980c
**Drinks**				
Beverages (alcoholic)	–	Bottled/canned	0.40–2.84	Powell et al., 2005
Tea (leaves/tea bags)	–	Black tea	0.81–0.86	Powell et al., 2005
Pineapple juice	–	Fresh juice	1.41	Powell et al., 2005
Coffee		Caffeinated	0.59	Powell et al., 2005
Water	–	Mineral, still	0.54	Powell et al., 2005
Orange juice	–	Fresh juice	0.13	Powell et al., 2005
Apple juice	–	Fresh juice	0.05–1	Varo et al., 1980c; Powell et al., 2005
**Others**				
Pharmaceuticals	–	–	Variable	Powell et al., 2005
Dust	–	–	NA	Powell et al., 2005
Cosmetics	–	–	NA	Powell et al., 2005

*NA, no data available.*

## Dietary Silicon Sources Reveal Major Contribution of Plants

Ubiquitous presence of Si in soil and plants provide the major dietary source of silica to the human body (**Figure 1d**). Si is the most abundant trace element in humans after Fe and Zn (Dobbie and Smith, 1982; Solomons, 1984; Wedepohl, 1995). The water soluble forms of silica such as orthosilicic acid is the main source for absorbed Si in humans and is associated with several health benefits related to structure and function of blood vessels, bones, kidney, liver, skin, and tendons etc. (Reffitt et al., 2003; Powell et al., 2005; Jugdaohsingh, 2007; Nielsen, 2014) (**Figure 1e**). As in plants, Si nutrition imparts several growth benefits particularly under stress conditions, but its biological role still remains an enigma. Si is still not considered as essential element despite accumulating evidence revealing a strong link between Si deficiency and bone deformities, reduced collagen contents, joint problems and improper mineral balance in femur and vertebrae (Carlisle, 1972; Schwarz and Milne, 1972; Seaborn and Nielsen, 2002). Major dietary Si requirement of human body is fulfilled by cereals such as rice, wheat, oat and barley (30%), followed by fruits (particularly banana and apple), vegetables (e.g., potato, beet roots, carrot, green beans, and reddish), beverages (alcoholic, hot, and cold combined) and some nuts and dried fruits such as raisins etc. (Pennington, 1991; Jugdaohsingh, 2007). Collectively, these foods provide >75% of the dietary Si intake (McNaughton et al., 2005). However, it is noteworthy that refinement of grains remove Si during the process but silica-derived food additives can replace the stripped Si and increase the content (Pennington, 1991). However, grain products such as breakfast cereals, rice, cake, biscuits, pasta, flour, and bread etc., are still high dietary sources of Si (Varo et al., 1980a,b,c; Pennington, 1991; Powell et al., 2005; **Table 1**). Other sources of Si include animal and fish meat, milk, eggs (Nielsen, 1974; Nuurtamo et al., 1980; Varo et al., 1980c; Bowen and Peggs, 2006), drinking water, fruit juices, alcoholic beverages, and even many pharmaceutical products such as capsules, gels, solutions, and tablets contain Si as supplement such as aluminum and magnesium silicates (Lomer et al., 2004; Powell et al., 2005). Several other products of daily use such as tooth paste, cosmetics, creams, and shampoos also contain Si but rather in inactive form. In some other cases, exposure to Si via dust and soil adhered to vegetables also fulfill silica requirement, but to minor extent due to low digestibility (Jugdaohsingh, 1999; Jugdaohsingh et al., 2002). As reported above, seed grains of cereals contain very high levels of silica, e.g., breakfast cereals and beer prepared from barley malt (Pennington, 1991; Sripanyakorn et al., 2004). But silica levels in dietary products decrease during industrial processing and along with the increasing trend to growing vegetables in hydroponic media omitting Si addition (Epstein, 1994; Sripanyakorn et al., 2004). Similarly, Si levels in drinking water vary with water source geology and water treatment processes reduce soluble Si contents (Perry and Keeling-Tucker, 1998). Daily Si intake can be categorized based on several reports from different regions of the world which results in the ranking of USA < other Western countries < Japan < China < India (Teraoka et al., 1981; Chen et al., 1994; Anusuya et al., 1996; Jugdaohsingh et al., 2002). The very high silica intake per person and day in India might be due to the predominant rice diet which greatly depends on personal dietary habits.

## Significance of Silica in Human Nutrition

In the last four decades, numerous studies have reported the beneficial effects of Si for human health (**Figure 1e**). It is estimated that human daily intake of Si as silicic acid ranges from 9 to 14 mg, while intakes near 25 mg/d might promote bone health. Initial experiments about nutritional significance of Si for human health were performed by Schiano et al. (1979), who found a significant increase in trabecular bone volume by using monomethyl trisilanol as an external silica source. Recent epidemiological experiments have reported that Si is involved in several biochemical functions including bone and connective tissue metabolism. Si is necessary for biosynthesis of collagen and glycosaminoglycan which are required for organic bone matrix formation (Carlisle, 1988; Hott et al., 1993). Evidence of dietary silica intake and its subsequent absorption, transport, retention, and excretion indicates that Si levels are well regulated in humans. Jugdaohsingh et al. (2002) reported that use of silica enriched food and drinks increases its absorption as well as consumption by human body. About 41% of the absorbed silica from food is excreted in urine while its concentration in the blood serum remains constant (10–31 μg/dL). Over a range of dietary intake the major part is retained in connective tissues, including bone, skin, trachea, and tendon, with another fraction being transferred to the brain (Carlisle, 1997; Jugdaohsingh, 2007; Robberecht et al., 2009). Prolonged intake of diets low in silica causes skull and bone disabilities in humans (Carlisle, 1981). Likewise, low Si levels in drinking water increase the risk of cognitive impairment due to high aluminum (Al) intake (Jacqmin-Gadda et al., 1996). Silicic acid forms complexes with aluminum hydroxide. The resulting aluminosilicates decrease the availability of free Al, hence prevents the occurrence of neurodegeneration in the brain (Domingo et al., 2011). In order to prevent risks of developing Al-induced Alzheimer’s disease, use of silica rich water with concentrations ≥11 mg/L is recommended (Gillette-Guyonnet et al., 2005). The presence of Si increases the absorption and utilization of other mineral elements such as magnesium and copper (Emerick and Kayongo-Male, 1990; Kikunaga et al., 1991). Additionally, Henrotte et al. (1988) suggested that Si plays a role in regulating the cell cycle of lymphocytes which ultimately affect the immune and inflammatory response. As in plants, Si is involved in signal transduction because it binds to hydroxyl groups of proteins (Rezanka and Sigler, 2007). It may be assumed that similar mechanisms of action improve mental health, immune and inflammatory response, and gene expression of factors involved in osteoblastogenesis and osteoclastogenesis.

## Conclusion and Future Prospects

The beneficial action of Si on cell physiology and stress acclimation-related processes is well established. Independent principle mechanisms likely contribute to the positive effects of Si: (i) Si at high concentrations changes physicochemical properties which affect solubility, binding and sequestration of other elements *ex planta* and *in planta*. Examples of this kind of effect have been described above. The challenge is to transfer and predict such processes for the cellular environment. More detailed subcellular compartmentation analyses combined with modeling of speciation may provide access to better understanding such processes. (ii) Si may bind to proteins such as effector proteins and receptors or compete with other binding processes in the cell or on the cell surface. The proof of concept comes from synthetic 12-mer peptides that could be selected from a phage display screening and efficiently bound to silicon surfaces (Estephan et al., 2011). Likewise the existence of Si transporters demonstrates specificity of silica recognition and transport (Ma et al., 2006, 2007b; Yamaji et al., 2008). Thus it appears timely to initiated Si metallomics screenings to identify high affinity Si binding entities and to analyze the role of identified candidates. (iii) Interference of Si with other cell processes by virtue of its high concentrations but with low affinity unlikely will be accessible by proteomic search for Si binding partners. Identification of indirect effects using omics technologies such as RNA profiling may be expected to provide circumstantial evidence for involved processes. Here a new type of experiment needs to be considered where stressed plants are supplemented with beneficial Si. The kinetics of the cell and tissue recovery responses at high time resolution may provide novel insight and dissect the order of involved processes. In rice addition of Si to Cd-stressed plants revealed full recovery of the stressed plant and separated fast and slow processes proving the potential of kinetic recovery experiments. These three strategies should also be employed for animal and human cell lines and experimental systems with vertebrates in order to improve our understanding of Si-dependent signaling and regulation in vertebrates.

Further, the increasing evidence of significance of silica nutrition for human health, mainly contributed by plant-based foods, suggests timely initiation of new concept of Si-bio-fortification of crop plants. Despite being among the most abundant elements on earth, silicates do not provide bioavailable dietary Si. In fact, phytolithic silica occurring in plants is often associated with polysaccharide/carbohydrate components of the cell wall and is only absorbed at 1–20% depending on the food source (Martin, 2007). Further, high levels of Si are found in unrefined grains such as wheat, oat, rice, and barley bran (Jugdaohsingh, 2007). Therefore, in order to improve the silica contents in the edible plant parts (Si-biofortification), two strategic approaches are proposed, namely (i) increasing silica bioavailability by reducing the anti-nutritional factors and favoring or increasing expression of nutritional factors. In recent years, the scientific community has published several studies related to the possibility of enriching micronutrients in the plant, using soil-less system as a tool for biofortification; (ii) enhancing nutritional quality of plant-based foods by applying molecular breeding tools as a strategy for modifying the content of silica in the edible parts of the plant.

A word of caution is needed, when just looking on a single trait like Si accumulation in context of improvement of Si nutrition in food and feed: Si transporters facilitate arsenite transport (Moore et al., 2011). Arsenite accumulation is not a preferable trait due to its toxicity to animals and humans. Si transporters are receiving attention since downregulation of Lsi transporters might be exploited as strategy to reduce As accumulation in rice (Moore et al., 2011). This example shows the delicate balance in nutrient homeostasis, here between Si enrichment and As avoidance, and that any change in single components intended to improve performance should be considered for possible side effects under other environmental conditions.

## Conflict of Interest Statement

The authors declare that the research was conducted in the absence of any commercial or financial relationships that could be construed as a potential conflict of interest.
